# Randomized Crossover Trial of Phosphate-binding Medication on Serum Phosphate Levels in Patients With Aortic Stenosis

**DOI:** 10.1016/j.clinthera.2019.08.004

**Published:** 2019-10

**Authors:** David S. Wald, John Chambers, Jonathan P. Bestwick, Nicholas J. Wald

**Affiliations:** 1 Wolfson Institute of Preventive Medicine, Barts and the London School of Medicine and Dentistry, Queen Mary University of London, London, United Kingdom; 2 Department of Cardiology, St Thomas Hospital, London, United Kingdom

**Keywords:** aortic stenosis, phosphate, phosphate-binding, prevention, sevelamer

## Abstract

**Purpose:**

Aortic stenosis is a common cause of valvular heart disease with no means of prevention. The recognized association between aortic stenosis and serum phosphate raises the possibility of preventing progression of the disorder by using phosphate-binding drugs, but there is uncertainty whether such treatment lowers serum phosphate levels in patients without diagnosed renal failure. This pilot study was conducted to answer this question in patients with aortic stenosis.

**Methods:**

A randomized, double-blind, crossover trial of the phosphate-binding drug sevelamer was conducted in 72 patients. Patients were prescribed sevelamer 0.8 g (low-dose), sevelamer 2.4 g (high-dose), and matching placebo, 3 times daily with food; each regimen lasted 6 weeks and was allocated at random. Serum phosphate levels were measured at the end of each treatment period, and within-person levels were compared.

**Findings:**

Sixty-one patients completed the 3 treatment periods. There was no significant difference in the mean end-treatment phosphate levels across all patients (3.38, 3.36, and 3.31 mg/dL with placebo, low-dose sevelamer, and high-dose sevelamer, respectively). Post hoc analysis showed a reduction in phosphate levels with increasing sevelamer dose in the highest baseline phosphate quartile group; a 0.3 mg/dL reduction (mean, 4.09 mg/dL with placebo, 3.95 mg/dL with low-dose sevelamer, and 3.79 mg/dL with high-dose sevelamer; *P*_trend_ = 0.027).

**Implications:**

Sevelamer had no overall statistically significant effect in lowering serum phosphate levels, but a reduction was observed in patients with phosphate levels in the highest quartile group of the population distribution. This hypothesis-generating result requires confirmation in an independent study. If confirmed, a trial of sevelamer in preventing the progression of aortic stenosis may be justified in patients with high phosphate levels. ISRCTN Registry identifier: ISRCTN17365679.

## Introduction

Aortic stenosis affects ~3% of people aged >75 years^1^, 2. and is the most common indication for heart valve replacement and cause of death from valvular heart disease in Western countries.^3^, ^4^ Calcium phosphate crystals accumulate on the aortic valve,^5^ leading to progressive obstruction of blood flow from the heart and death in symptomatic cases, unless the valve is surgically replaced.^6^ There is no known means of prevention.

Observational studies have reported increased serum phosphate levels, within the population range of values, in patients with aortic stenosis; a meta-analysis found that a 0.1 mmol/L (0.31 mg/dL) increase in serum phosphate (about a 10% increase in usual levels) was associated with an ~50% increase in the risk of aortic stenosis.^7^ This finding suggests that reducing serum phosphate levels could reduce calcium phosphate accumulation on the valve and slow or arrest progression of the disorder.

Phosphate-lowering drugs such as sevelamer, which bind dietary phosphate in the gut, are used in patients with renal failure undergoing dialysis to lower raised serum phosphate levels, typically >5.5 mg/dL, but it is not known whether such treatment lowers serum phosphate levels in people not on dialysis. It is uncertain whether treatment, in the absence of diagnosed renal failure, will lower phosphate levels because preserved renal function may counter the effect of reduced dietary phosphate absorption.^8^ This uncertainty prompted us to conduct a randomized, double-blind, placebo-controlled crossover trial to determine whether sevelamer reduces serum phosphate levels in patients without diagnosed renal failure. We selected individuals with aortic stenosis to directly examine the treatment effect in such patients, because the effect may differ from that in others, a possibility that might arise because of the tendency for patients with aortic stenosis to have raised phosphate levels.^7^ Also, the relevance of the trial to their disorder was likely to enhance adherence to treatment.

## Patients and Methods

Participants for this randomized crossover trial were enrolled between June 2017 and June 2018 from cardiology clinics or an echocardiography database at 2 London cardiac centers, St. Bartholomew's Hospital and Guy's and St Thomas Hospitals. All had mild to moderate aortic stenosis defined as a peak velocity of trans-aortic valve blood flow between 2.0 and 4.0 m/s. They were not eligible if they were: pregnant or breastfeeding; allergic to sevelamer; had a history of hypophosphatemia, bowel obstruction, or lactose intolerance; required phosphate-binding drugs for other reasons or took drugs that interact with phosphate-binding drugs; or had any illness that in the judgment of the supervising physician contraindicated participation. The study was approved by the London–Westminster Research Ethics Committee, part of the UK national system. Participants provided written informed consent before participation.

**Figure 1:**
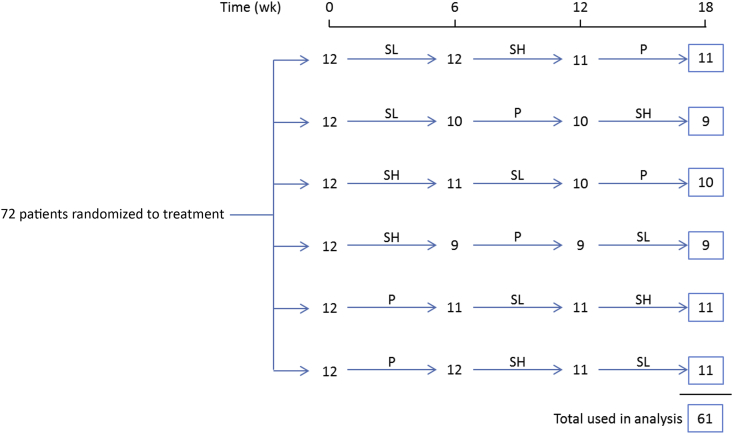
Flow diagram of participants with aortic stenosis in the trial. A total of 494 patients were screened; 59 were ineligible, 219 declined to participate, and 144 did not respond (leaving 72 patients). P = placebo; SH = high-dose sevelamer (2.4 g per meal); SL = low-dose sevelamer (0.8 g per meal).

Trial participants were asked to take treatment 3 times daily with meals for 6 weeks (sevelamer 0.8 g per meal [low-dose], sevelamer 2.4 g per meal [high-dose], and a matching placebo) in a crossover trial design. The sequence of treatments was allocated at random (Figure 1).

The randomization sequence was generated in advance by the study statistician using a computer random number generator in blocks of 6. Blood and urine samples were collected at the end of each 6-week period (usually between 8:00 am and 10:00 pm, after an overnight fast). Six weeks of treatment or placebo was judged sufficient time for the drug in the previous period to have “washed out” because it is known that phosphate levels return to pretreatment levels after 2 weeks of stopping sevelamer treatment.^9^

Sufficient medication was provided for 3 meals per day, with a maximum daily dose of 2.4 g in the low-dose period and 7.2 g in the high-dose period. Patients were advised to omit treatment if a meal was missed. The tablets were packaged in daily blister strips providing 3 pills per meal, using placebo pills to make the total number of pills up to 3 in the lower dose period. In this way, an equal number of identical pills were taken with each meal in each treatment period. Neither the patients nor the investigators knew the sequence. No dietary modification was required during the trial; the only systematic intervention was the treatment taken (sevelamer or placebo).

Venous blood samples were collected and serum phosphate concentration assayed within 48 h of collection by using the colorimetric reaction method with ammonium molybdate (%CV, 0.8%).^10^ Urine samples were assayed within 48 h by using the same method for phosphate and the Jaffe colorimetric method for creatinine (%CV, 2.5%),^11^, ^12^ the 2 measurements used together with age and weight to estimate 24-h urine according to the method of Robinson-Cohen et al.^13^ Biochemical analyses were performed “blind” (ie, without knowing which samples followed treatment with sevelamer or placebo). At the end of each 6-week treatment period, patients completed a questionnaire on recognized side effects of sevelamer (bloating, nausea, reflux, diarrhea, abdominal pain, vomiting, constipation, or rash) and answered an open question on any other symptoms that had arisen.

To encourage adherence to treatment, participants were sent text message reminders before each meal during the first week of treatment and at reduced intervals thereafter. If a meal was not eaten, the patient was told not to take the treatment and was asked to mark this on the blister strip against the pills not taken. Participants were asked to return all empty and unused blister strips at the end of each 6-week treatment period and adherence was assessed using pill counts, allowing for pills not taken because meals were not eaten. Urine phosphate measurements also provided a measure of adherence.

With 72 participants, the trial had 80% power to show a statistically significant (*P* < 0.05) effect on serum phosphate levels assuming a mean reduction in serum phosphate of at least 0.5 mg/dL, allowing for a 25% noncompletion rate. Patients who completed the trial were included in the statistical analysis of efficacy based on within-person measurements at the end of each treatment period using paired *t* tests.

A post hoc analysis of treatment effect stratified according to quartiles of baseline serum phosphate was performed. The prevalence of adverse effects using data on all patients was assessed including why patients who did not complete all 3 periods discontinued the study. A repeated measures analysis of variance was also performed as a check to determine whether there was any evidence in the data of 3 sources of error that can arise in crossover trials: (1) period effects (variable measured changes in participants during the course of the trial regardless of intervention); (2) sequence effects (when the order of interventions affects the result); and (3) carryover effects (when the effect of an intervention in a previous period persists in a subsequent period). The analysis used STATA pkcross command tests against the “null hypothesis” (expressed as a *P* value). STATA version 14 (StataCorp, College Station, Texas) was used for all statistical analyses.

## Results

Of 494 patients screened, 72 were enrolled in the study. The Table shows the characteristics of the 72 patients (40 from St. Bartholomew's Hospital and 32 from Guy's and St Thomas' Hospitals). Eleven participants did not complete the trial (Figure 1); the main analysis was therefore based on the 61 patients completing all 3 treatment periods.

**Table:** Characteristics of 72 patients with aortic stenosis enrolled in the crossover trial.

Characteristic	Value
Age, range, y	65 (35–88)
No. of men	53
No. of women	19
Height, m∗	1.72 (0.10)
Weight, kg∗	83.5 (18.1)
Echocardiography	
Peak trans-aortic velocity, m/s∗	2.8 (0.60)
Peak trans-aortic pressure gradient, mm Hg∗	33 (10)
Baseline biochemistry	
Serum phosphate, mg/dL∗	3.30 (0.52)
Serum creatinine, mg/dL†	1.0 (0.84–1.21)
eGFR, mL/min/1.73 m^2^†	74 (60–88)

eGFR = estimated glomerular filtration rate.

∗ Mean (SD).

† Median (interquartile range).

**Figure 2:**
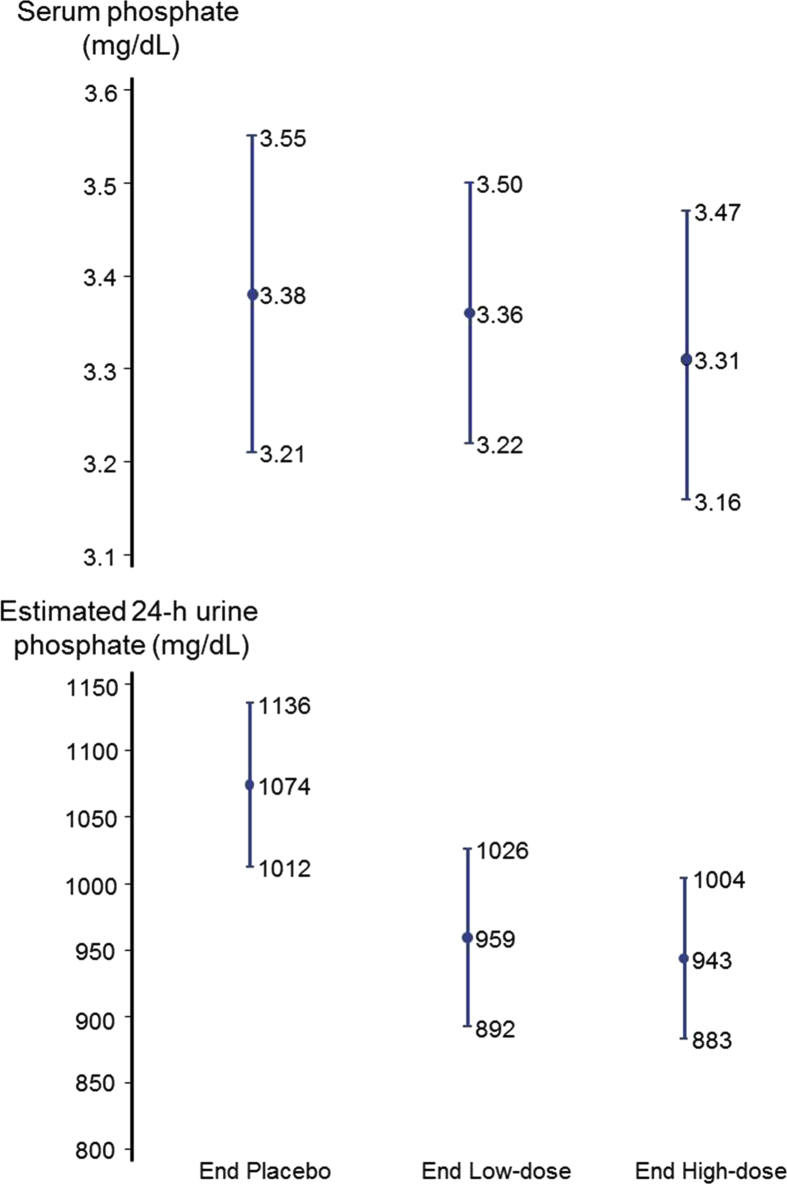
Mean of within-person serum phosphate and estimated 24-h urine phosphate levels (95% CIs) at the end of the placebo period, end of the low-dose (0.8 g per meal) sevelamer period, and end of the high-dose (2.4 g per meal) sevelamer period. *P* values were not significant for serum phosphate for either low-dose or high-dose sevelamer versus placebo and *P* < 0.001 for urine phosphate for both low-dose and high-dose sevelamer versus placebo.

Figure 2 shows the mean serum phosphate results at the end of the 3 study periods (placebo, low-dose sevelamer, and high-dose sevelamer). There was a suggestion of a reduction in serum phosphate at the end of the sevelamer periods (greater with high-dose sevelamer than with low-dose sevelamer), but this finding was not statistically significant (*P* values for low-dose sevelamer vs placebo, *P* = 0.720; high-dose sevelamer vs placebo, *P* = 0.306; and *P*_trend_ = 0.807). Estimated 24-h urine phosphate measurements revealed reductions in excretion of phosphate during the low-dose and high-dose sevelamer periods (*P* < 0.001 for each dose compared with placebo). In the analysis of variance, there was no indication of a sequence effect (*P* = 0.954), a period effect (*P* = 0.718), or a carryover effect (*P* = 0.764) on serum phosphate levels, and the point estimates were virtually identical to those shown in Figure 2.

**Figure 3:**
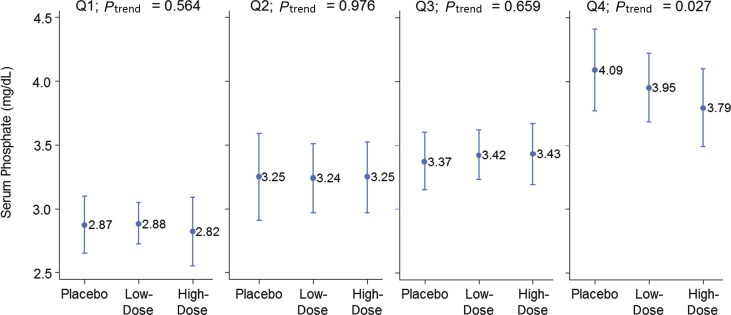
Post hoc analysis of the mean (95% CI) end-of-treatment levels of serum phosphate stratified according to baseline serum phosphate quartile (Q) groups. Q1 = 2.20–2.94 mg/dL; Q2 = 2.95–3.28 mg/dL; Q3 = 3.29–3.62 mg/dL; Q4 = 3.63–4.77 mg/dL; High-dose = 2.4 g of sevelamer per meal; Low-dose = 0.8 g of sevelamer per meal.

Figure 3 shows the results of a post hoc analysis of the mean end-of-treatment levels of serum phosphate stratified according to baseline serum phosphate quartile groups (2.20–2.94, 2.95–3.28, 3.29–3.62, and 3.63–4.77 mg/dL for the first, second, third, and fourth quartile groups, respectively). In the fourth (highest) quartile group, there was a statistically significant trend in reducing serum phosphate levels with increasing sevelamer dose (serum phosphate was 4.09 mg/dL with placebo, 3.95 mg/dL with low-dose sevelamer, and 3.79 mg/dL with high-dose sevelamer; *P*_trend_ = 0.027); this is about a 0.3 mg/dL reduction in serum phosphate level using high-dose sevelamer. Renal function was not significantly different in the highest phosphate quartile group compared with the lowest quartile groups (estimated glomerular filtration rate, 69 vs 74 mL/min/1.73 m^2^). Similarly, there were no significant differences in urine phosphate levels (930 vs 1020 mg/dL).

Eleven patients did not complete all 3 treatment periods: 7 while taking high-dose sevelamer (2 with constipation, 1 with bloating, 1 on warfarin with a high international normalized ratio, and 3 gave no reason), 3 patients while taking low-dose sevelamer (1 said there were too many pills to take, and 2 gave no reason), and 1 patient while taking placebo (too many pills to take) (*P* = 0.03 high-dose sevelamer vs placebo). There were no statistically significant differences in the number of patients reporting specified symptoms at the end of each treatment period (bloating, nausea, diarrhea, abdominal pain, vomiting, constipation, rash, reflux, and other); 25, 23, and 28 patients reported one or more adverse events with placebo, low-dose sevelamer, and high dose sevelamer, respectively. Adherence to treatment was high: 90% of patients took at least 90% of their prescribed pills. The mean daily doses of sevelamer, taking into account meals not eaten, during the low- and high-dose periods were 2.33 g/d and 6.95 g/d.

## Discussion

This pilot trial found no overall statistically significant effect of sevelamer in reducing serum phosphate levels. This outcome is not explained by poor adherence to treatment given the data on pill counts and urine phosphate levels showing reduced phosphate excretion. A post hoc analysis revealed a statistically significant reduction in serum phosphate levels among patients in the highest quartile subset of the population.

It is well recognized that a post hoc analysis can lead to a formally statistically significant result that could readily have been due to chance. At the same time, such significant results can reasonably be used in a “hypothesis-generating” way, encouraging further studies to see if the finding can be confirmed.

If the top quartile subset analysis is confirmed, there is then the prospect of extending the use of sevelamer beyond its licensed indication (control of hyperphosphatemia in patients with end-stage renal disease undergoing dialysis) to patients without renal failure, where it may have a role in preventing the progression of aortic stenosis. Three previous trials have examined the use of sevelamer on serum phosphate in patients not undergoing dialysis.^14^, ^15^, ^16^ One was small and of short duration (12 healthy volunteers treated for 6 days),^14^ showing no significant effect, and two were based on patients diagnosed with moderate renal failure (chronic kidney disease stage 3); one of these showed no significant effect^15^ and the other a 0.2 mg/dL reduction,^16^ similar to our result in the highest quartile group. Our trial could have been undertaken in any patient group without diagnosed renal failure, but conducting the trial in patients with aortic stenosis had the advantage of high treatment adherence, because, as expected, patients with aortic stenosis are likely to be motivated given the relevance of the research to their disorder; this was the case in the present study.

If the suggestion is confirmed that sevelamer lowers high phosphate levels with little or no effect at low levels, it would be another example of treatment effect on physiological variables tending to be greater when the variable is high than when it is low (eg, the effect of blood pressure–lowering therapy on blood pressure).^17^ The hypothesis that a phosphate-binding drug such as sevelamer reduces high phosphate levels could be tested in a clinical efficacy study, with echocardiographic assessment of aortic stenosis as the end point. A possible research strategy could involve a parallel group trial based on a larger sample of patients limited to individuals with serum phosphate levels >3.65 mg/dL (the top quartile group in our trial) using the dose of 2.4 g per meal. An interim analysis could be undertaken to determine whether the reduction in serum phosphate level observed here is confirmed and, if so, the trial could be continued to examine an effect on the rate of progression of aortic stenosis. If the positive association between serum phosphate and aortic stenosis shown in observational studies^7^ is causal and reversible, and if the results of our trial based on the patients with high serum phosphate levels are real, sevelamer would be a useful treatment to prevent aortic stenosis progression, which is the ultimate clinical objective.

The crossover design, in which each person was their own control, is statistically powerful, and the hypothesis-generating result in patients with a high serum phosphate level is one that might have otherwise been missed. Only further research will show whether the result is confirmed. Crossover trials often include washout periods between treatments, but this is not necessary if the treatment periods are long enough to exclude carryover from a previous treatment and comparisons are performed on outcome measurements made at the end of each treatment period.^18^, ^19^ It has been shown that a 2-week period is long enough for the effects of sevelamer to return to baseline after stopping treatment,^12^ three times less than the 6-week treatment periods in our trial. We have used this method before^20^; it has the advantage of reducing demands on patients by requiring fewer patient visits (4 visits, instead of 6 visits, in this trial).

If lowering serum phosphate levels delays the progression of aortic stenosis, patients may be willing to accept the treatment regimen long term, but loss of bone density resulting from phosphate reduction is a concern. This was not an outcome in our study, but it is reassuring that in 2 sevelamer trials in patients with moderate to severe renal failure, there was no reduction in bone density (assessed by computed tomography scanning of the lumbar spine) after ~40 weeks of treatment.^15^, ^16^ A long-term study of sevelamer on the prevention of aortic stenosis could usefully examine the effect on bone density and fractures. The present study did not assess the effect of sevelamer on preventing the progression of aortic stenosis, but it does provide information to help guide the future research needed to determine whether this is possible through phosphate reduction.

## Conclusions

Sevelamer had no overall statistically significant effect in lowering serum phosphate levels, but a reduction was observed in patients with high phosphate levels. This hypothesis-generating result requires confirmation in an independent study.

## Figures and Tables

**Table:** 

Section/Topic	Item No	Checklist item (Prevention of Aortic Stenosis Pilot Trial)	Reported on page No
**Title and abstract**			
	1a	Identification as a randomised trial in the title	1
	1b	Structured summary of trial design, methods, results, and conclusions (for specific guidance see CONSORT for abstracts)	2
**Introduction**			
Background and objectives	2a	Scientific background and explanation of rationale	3
	2b	Specific objectives or hypotheses	3
**Methods**			
Trial design	3a	Description of trial design (such as parallel, factorial) including allocation ratio	4
	3b	Important changes to methods after trial commencement (such as eligibility criteria), with reasons	N/A
Participants	4a	Eligibility criteria for participants	4
	4b	Settings and locations where the data were collected	4
Interventions	5	The interventions for each group with sufficient details to allow replication, including how and when they were actually administered	4,5
Outcomes	6a	Completely defined pre-specified primary and secondary outcome measures, including how and when they were assessed	4,5,6
	6b	Any changes to trial outcomes after the trial commenced, with reasons	N/A
Sample size	7a	How sample size was determined	6
	7b	When applicable, explanation of any interim analyses and stopping guidelines	N/A
Randomisation:			
Sequence generation	8a	Method used to generate the random allocation sequence	4
	8b	Type of randomisation; details of any restriction (such as blocking and block size)	4
Allocation concealment mechanism	9	Mechanism used to implement the random allocation sequence (such as sequentially numbered containers), describing any steps taken to conceal the sequence until interventions were assigned	4
Implementation	10	Who generated the random allocation sequence, who enrolled participants, and who assigned participants to interventions	4
Blinding	11a	If done, who was blinded after assignment to interventions (for example, participants, care providers, those assessing outcomes) and how	4
	11b	If relevant, description of the similarity of interventions	4
Statistical methods	12a	Statistical methods used to compare groups for primary and secondary outcomes	5,6
	12b	Methods for additional analyses, such as subgroup analyses and adjusted analyses	5,6
**Results**			
Participant flow (a diagram is strongly recommended)	13a	For each group, the numbers of participants who were randomly assigned, received intended treatment, and were analysed for the primary outcome	7,8
	13b	For each group, losses and exclusions after randomisation, together with reasons	7
Recruitment	14a	Dates defining the periods of recruitment and follow-up	7
	14b	Why the trial ended or was stopped	7
Baseline data	15	A table showing baseline demographic and clinical characteristics for each group	Table 1
Numbers analysed	16	For each group, number of participants (denominator) included in each analysis and whether the analysis was by original assigned groups	7, Fig 2
Outcomes and estimation	17a	For each primary and secondary outcome, results for each group, and the estimated effect size and its precision (such as 95% confidence interval)	7, Fig 2
	17b	For binary outcomes, presentation of both absolute and relative effect sizes is recommended	N/A
Ancillary analyses	18	Results of any other analyses performed, including subgroup analyses and adjusted analyses, distinguishing pre-specified from exploratory	7, Figure 3
Harms	19	All important harms or unintended effects in each group (for specific guidance see CONSORT for harms)	8
**Discussion**			
Limitations	20	Trial limitations, addressing sources of potential bias, imprecision, and, if relevant, multiplicity of analyses	9
Generalisability	21	Generalisability (external validity, applicability) of the trial findings	9,10
Interpretation	22	Interpretation consistent with results, balancing benefits and harms, and considering other relevant evidence	9,10,11
**Other information**			
Registration	23	Registration number and name of trial registry	2
Protocol	24	Where the full trial protocol can be accessed, if available	11
Funding	25	Sources of funding and other support (such as supply of drugs), role of funders	11

*We strongly recommend reading this statement in conjunction with the CONSORT 2010 Explanation and Elaboration for important clarifications on all the items. If relevant, we also recommend reading CONSORT extensions for cluster randomised trials, non-inferiority and equivalence trials, non-pharmacological treatments, herbal interventions, and pragmatic trials. Additional extensions are forthcoming: for those and for up to date references relevant to this checklist, see www.consort-statement.org.

## Appendix A Supplementary data

**CONSORT 2010 checklist of information to include when reporting a randomised trial***
